# Antibodies Against Hypothalamus and Pituitary Gland in Childhood-Onset Brain Tumors and Pituitary Dysfunction

**DOI:** 10.3389/fendo.2020.00016

**Published:** 2020-02-18

**Authors:** Giuseppa Patti, Erika Calandra, Annamaria De Bellis, Annalisa Gallizia, Marco Crocco, Flavia Napoli, Anna Maria Elsa Allegri, Hanan F. Thiabat, Giuseppe Bellastella, Maria Ida Maiorino, Maria Luisa Garrè, Stefano Parodi, Mohamad Maghnie, Natascia di Iorgi

**Affiliations:** ^1^Department of Pediatrics, IRCCS Istituto Giannina Gaslini, University of Genova, Genova, Italy; ^2^Department of Neuroscience, Rehabilitation, Ophtalmology, Genetics, Maternal and Child Health, University of Genova, Genova, Italy; ^3^Department of Advanced Medical and Surgical Sciences Endocrinology and Metabolic Unit, University of Campania Luigi Vanvitelli, Naples, Italy; ^4^Department of Pediatrics, IRCCS Istituto Giannina Gaslini, Genoa, Italy; ^5^Neurosurgery Unit, IRCCS Istituto Giannina Gaslini, Genoa, Italy; ^6^Epidemiology and Biostatistics Unit, IRCCS Istituto Giannina Gaslini, Genoa, Italy

**Keywords:** pituitary, brain tumor, germinoma, growth hormone deficiency, autoimmunity, diabetes insipidus

## Abstract

**Purpose:** To detect the presence of antipituitary (APA) and antihypothalamus antibodies (AHA) in subjects treated for brain cancers, and to evaluate their potential association with pituitary dysfunction.

**Methods:** We evaluated 63 patients with craniopharyngioma, glioma, and germinoma treated with surgery and/or radiotherapy and/or chemotherapy at a median age of 13 years. Forty-one had multiple pituitary hormone deficiencies (MPHD), six had a single pituitary defect. GH was the most common defect (65.1%), followed by AVP (61.9%), TSH (57.1%), ACTH (49.2%), and gonadotropin (38.1%). APA and AHA were evaluated by simple indirect immunofluorescence method indirect immunofluorescence in patients and in 50 healthy controls.

**Results:** Circulating APA and/or AHA were found in 31 subjects (49.2%) and in none of the healthy controls. In particular, 25 subjects out of 31 were APA (80.6%), 26 were AHA (83.90%), and 20 were both APA and AHA (64.5%). Nine patients APA and/or AHA have craniopharyngioma (29%), seven (22.6%) have glioma, and 15 (48.4%) have germinoma. Patients with craniopharyngioma were positive for at least one antibody in 39.1% compared to 33.3% of patients with glioma and to 78.9% of those with germinoma with an analogous distribution for APA and AHA between the three tumors. The presence of APA or AHA and of both APA and AHA was significantly increased in patients with germinoma. The presence of APA (*P* = 0.001) and their titers (*P* = 0.001) was significantly associated with the type of tumor in the following order: germinomas, craniopharyngiomas, and gliomas; an analogous distribution was observed for the presence of AHA (*P* = 0.002) and their titers (*P* = 0.012). In addition, we found a significant association between radiotherapy and APA (*P* = 0.03).

**Conclusions:** Brain tumors especially germinoma are associated with the development of hypothalamic–pituitary antibodies and pituitary defects. The correct interpretation of APA/AHA antibodies is essential to avoid a misdiagnosis of an autoimmune infundibulo-neurohypophysitis or pituitary hypophysitis in patients with germinoma.

## Introduction

Hypothalamic–pituitary (HP) dysfunction in subjects with intracranial mass involving the sellar and parasellar region may be due to several causes including the local compressive effect, inflammation, and or infiltration by the primary lesion, neurosurgery, and or radiotherapy (1). The differential diagnosis of HP tumors may be challenging despite a prompt biopsy because of lymphocyte infiltration of the central nervous system (CNS) tissues as a host reaction (2–4).

The interaction between brain tumors and the immune system was a field of intensive research in the last two decades leading to significant advances in the understanding of the pathobiology of these cancers as well as to successful immunological treatment strategies (5–8). On one side, the involvement of the innate immune response in craniopharyngioma cyst formation was reported after the detection of the antimicrobial peptides α-defensins 1–3 (9); on the other side, the evidence that CNS germinomas harbor germ cell components that stained positively for the programmed death ligand-1 expressed on tumor cells suggested that immune checkpoint inhibitors may have a therapeutic role in their future treatment (10).

Despite the fact that the neoplastic component of CNS germinoma is the germ cell, these tumors also exhibit an abundance of quiescent tumor-infiltrating lymphocytes (10). Hence, inflammatory lesions of the pituitary/pituitary stalk/hypothalamus including the chronic lymphoplasmacytic process known as lymphocytic infundibulo-hypophysitis, autoimmune hypophysitis, granulomatous inflammation, and xanthomatous hypophysitis (1, 11, 12) may raise diagnostic difficulties with tumors like germinomas. Both lymphocytic hypophysitis with central diabetes insipidus (CDI) and subsequent hypopituitarism masking a suprasellar germinoma, and isolated lymphocyte infiltration of pituitary stalk preceding the diagnosis of germinoma were reported (2–4). In addition, antipituitary antibodies (APA) and antihypothalamus (AHA) are often seen in patients with different pituitary pathologies including hypopituitarism, central hypothyroidism, acromegaly, adenomas, CDI, and in patients with Prader–Willi syndrome associated with hypothalamic dysfunction (13–21). Likewise, a mouse model of autoimmune hypophysitis showed endocrine dysfunction and disease progression associated with pituitary expansion followed by pituitary atrophy, thus suggesting the existence of a relationship between autoimmunity and endocrinopathy (22).

We hypothesized that the local immune cell response associated with HP brain tumors and their treatment modalities could lead to the development of autoantibodies, making the differential diagnosis with other conditions more challenging. In light of this hypothesis, the aim of this study was to detect the presence of APA and AHA in children and young adults with craniopharyngioma, glioma, or germinoma. We also intended to analyze their association with pituitary deficiencies.

## Patients and Methods

### Subjects

This is a cross-sectional single-center observational study conducted in children and adolescents with brain tumors at the Pediatric Clinic, Clinical Service of Endocrinology, Diabetes and Metabolism, and the Neurosurgery/Neuro-oncology unit, IRCCS Istituto Giannina Gaslini, University of Genova. Sixty-three patients (34 males and 29 females) with a median age of 13 years (IQR: 10–19) were evaluated at a median time of 5.5 years (IQR: 2.2–8.3) after the diagnosis of brain cancer when samples for the measurements of auto-antibodies were taken.

Intracranial tumors involving the HP region were craniopharyngiomas (*n* = 23), gliomas (*n* = 21), and germinomas (*n* = 19). Surgery was performed in 32 patients, radiotherapy in 46, and chemotherapy in 30 (Table 1). In particular, treatment combinations were as follows: surgery (*n* = 8); surgery and radiotherapy (*n* = 16); surgery, radiotherapy, and chemotherapy (*n* = 5); surgery and chemotherapy (*n* = 2); radiotherapy (*n* = 6); radiotherapy and chemotherapy (*n* = 19), chemotherapy (*n* = 4) (Table 1). Three subjects with glioma did not receive any treatment, and pituitary function was preserved at the time of the study in all of them. Forty-one subjects had multiple pituitary hormone deficiencies (MPHD), six had isolated hormone defect (growth hormone, GH, in five cases and vasopressin, AVP, in one case), 16 patients had preserved pituitary function (15 with glioma and one with germinoma). GH deficiency was the most common defect (65.1%), followed by AVP (61.9%), thyroid hormone deficiency (57.1%), adrenal insufficiency (49.2%), and hypogonadotropic hypogonadism (38.1%). Anterior pituitary function was assessed in all patients, both at the time of diagnosis and at the follow-up. Pituitary defects according to the type of brain tumor are reported in Table 1.

**Table 1:** Clinical characteristics and treatment of 63 patients with brain tumors according to the type of tumor.

	**Craniopharyngiomas** ***n* = 23** **Median, IQR**	**Gliomas** ***n* = 21** **Median, IQR**	**Germinomas** ***n* = 19** **Median, IQR**
Age at tumor diagnosis (years)	8.7 (4.8–10.0)^a^^,^^b^^,^^c^	3.5 (2.0–6.8)	11.5 (10.5–14.5)
Time between diagnosis and antibodies assessment (years)	7.0 (13.2–2.7)^d^	5.2 (2.2–6.8)	4.1 (1.4–1.7)
Pituitary defects at antibodies assessment	4 (3–5)^e^	0 (0–1)^b^	4 (3–5)
	**(*****n*****, %)**	**(*****n*****, %)**	**(*****n*****, %)**
AVPD	21 (91.3)^e^	1 (4.8)^b^	17 (89.5)
GHD	19 (82.6)^e^	5 (23.8)^b^	17 (89.5)
TSHD	20 (87.0)^e^	2 (9.5)^b^	14 (73.7)
ACTHD	17 (73.9)^e^	1 (4.8)^b^	13 (68.4)
GND	13 (56.5)^e^	1 (4.8)^g^	10 (52.6)
Males (*n* = 34)	12 (52.2)	10 (47.6)	12 (63.2)
Females (*n* = 29)	11 (47.8)	11 (52.4)	7 (36.8)
Surgery (*n* = 32)	22 (95.7)^e^^,^^f^	5 (23.8)	5 (26.3)
Radiotherapy (*n* = 46)	16 (69.6)	11 (52.4)	19 (100)^g^

*IQR, Interquartile Range*.

a *p < 0.01 Craniopahryngiomas vs. germinomas*.

b *p < 0.001 Germinomas vs. gliomas*.

c *p < 0.05 Craniopharyngiomas vs. gliomas*.

d *p = 0.088 Craniopharyngiomas vs. germinomas*.

e *p < 0.001 Craniopharyngiomas vs. gliomas*.

f *p < 0.001 Craniopahryngiomas vs. germinomas*.

g *p < 0.01 Germinomas vs. gliomas*.

*AVPD, vasopressin deficiency; GHD, growth hormone deficiency; TSHD, central hypothyroidism; ACTHD, central adrenal insufficiency; GND, hypogonadotropic hypogonadism*.

Basal anterior pituitary hormones and the respective target gland hormones were measured. Dynamic tests to evaluate anterior pituitary function were also performed. ACTH-D, GHD, and hypogonadism were diagnosed as previously described (15). In particular, ACTH-D, in the presence of normal or low basal serum levels of ACTH, was suspected when the 8.00 a.m. cortisol level was below 100 nmol/L (3.6 mcg/dl) and was confirmed by an impaired cortisol response to the 1-mcg tetracosactrin test (<440 nmol/L; 16 mcg/dl). GHD was diagnosed in the presence of clinical criteria (height velocity more than 2 SD below the mean over 1 year or more than 1.5 SD sustained over 2 years), low basal IGF-1, and impaired GH response to two stimulation tests (insulin-induced hypoglycemia, arginine, clonidine, glucagon). Hypogonadism was diagnosed in boys and girls who had no onset or progress of pubertal development and no increase in testosterone or estradiol levels as well as in FSH and LH after GnRH. Basal testosterone or estradiol, and FSH and LH, before and 15, 30, 60, and 120 min after the i.v., administration of 100 μg/m2 GnRH were measured in patients who were suspected to have hypogonadotropic hypogonadism. The pituitary–thyroid axis was assessed every 6–12 months by measuring serum FT4, FT3, and TSH. Hypothyroidism was defined as a low or low-normal serum TSH concentration and low serum FT4, FT3 concentration.

The diagnosis of central diabetes insipidus was suspected on the basis of evidence of polyuria, polydipsia, and urinary and plasma osmolality. To confirm the diagnosis of CDI, the patients underwent a dehydration test followed by a desmopressin administration test. A ratio of urinary osmolality to plasma osmolality of 1 or less was taken to indicate the presence of complete central diabetes insipidus, and a ratio of more than 1.0 but <1.4 was taken to indicate partial central diabetes insipidus. Moreover, an increase in urinary osmolality of more than 50%, or from 10 to 50%, after desmopressin injection allowed the diagnosis of complete or partial CDI, respectively.

The patients underwent these tests at the first observation. Subjects with pituitary deficiencies were receiving conventional replacement therapy: for GHD with rhGH, for thyroid hormones deficiencies with L-thyroxine, for adrenal insufficiency with hydrocortisone at a dose of 7–10 mg/m^2^/day, for hypogonadotropic hypogonadism with testosterone enantate, or ethinylestradiol/transdermal 17β-estradiol patches with medroxyprogesterone. Moreover, impaired AVP concentration was treated with desmopressin acetate (desamino-D-arginine-8-vasopressin, DDAVP). The study was approved by the ethics committee of Istituto Giannina Gaslini, Genova, and all patients or their tutors gave their informed consent to the study.

### Methods

**Figure 1:**
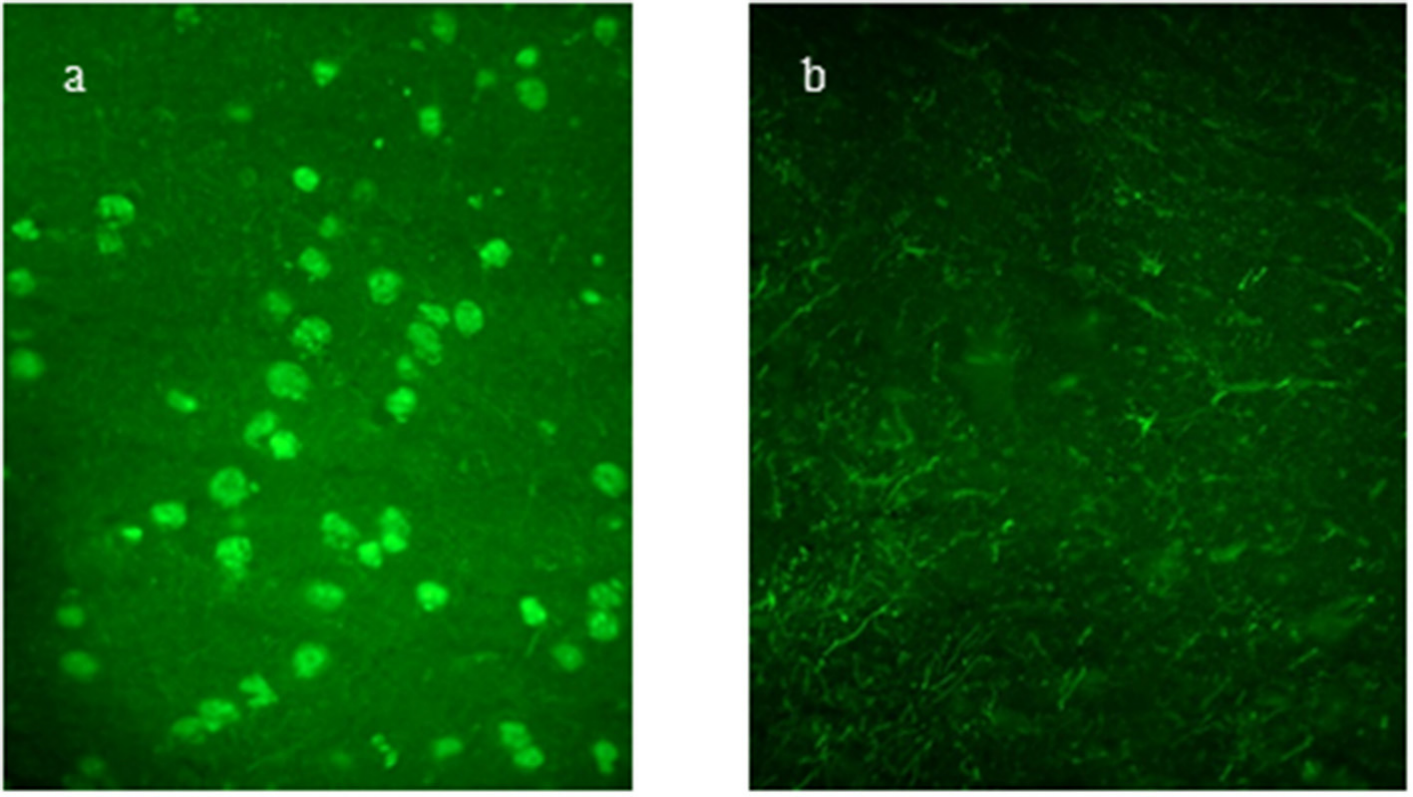
**(a,b)** Antipituitary antibodies (APA) detected by immunofluorescence method: positive serum sample showing intracytoplasmatic immunofluorescence of pituitary cells; negative control serum.

**Figure 2:**
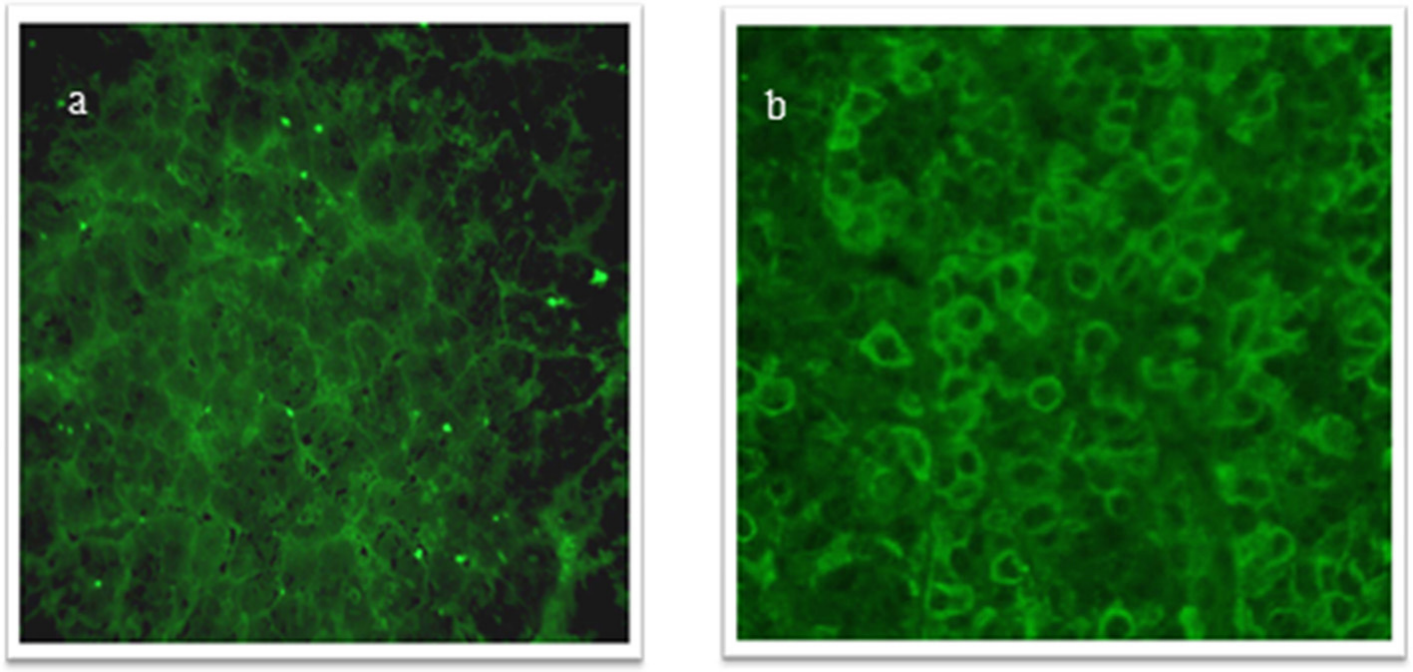
**(a,b)** Antihypothalamus antibodies (AHA) detected by immunofluorescence method: positive serum sample showing intracytoplasmatic immunofluorescence of hypothalamus cells; negative control serum.

Antipituitary antibodies and AHA were evaluated at the “Endocrinology and Metabolic Unit, Department of Advanced Medical and Surgical Sciences, University of Campania Luigi Vanvitelli, Naples” by a simple indirect immunofluorescence method on cryostat sections of young baboon pituitary gland and young baboon hypothalamus supplied by Halifax spa (Polverara, Pordenone, Italy) and Biomedis srl (Rome, Italy), respectively, as previously described (15, 16). In particular, unfixed cryostat sections of young normal baboon hypothalamus were initially incubated with the sera. Then, serum samples were subsequently tested with fluorescein isothiocyanate-conjugated goat antihuman Ig to detect the presence of APA and AHA. We considered APA and AHA positive serum samples starting at a dilution of 1:8; however, taking into account the low specificity of the immunofluorescence method, we considered for our analysis only the patients positive for APA and AHA at a high titer (>1:32). APA and AHA were also evaluated in the sera of 50 sex-/age-matched healthy persons. The control group (27 F, 23 M) was recruited from healthy childhood to transition age who had no other known causes of pituitary dysfunction; moreover, none of them had an individual or familial history of autoimmune diseases. APA was evaluated by two different operators in a double-blind manner (16, 17). The collaborators performing the immunological evaluation were also blinded to the pituitary deficiency status of the patients (18). Figures 1a,b show a sample positive for APA, and Figures 2a,b a sample positive for AHA by immunofluorescence, compared to two negative control samples.

### Statistical Analysis

Descriptive data were expressed as median and interquartile range (IQR), due to the non-normal distribution of most considered variables. Accordingly, differences between groups were analyzed by the non-parametric Mann–Whitney U test or the Kruskal–Wallis test in the presence of more than two groups. Comparisons between categorical or nominal variables were performed by the chi-squared test or the Fisher exact test (where appropriate). The Poisson regression model was employed to analyze the number of deficits by group (0, 1, 2, 3, 4, 5 defects). Its association with the AHA and APA titers was estimated by calculating the mean difference percent (MDP) between contiguous APA/AHA levels, by applying the following equation: *MDP* = (*e*^β^ − 1) × 100 where β represents the coefficient of the Poisson model. The same analyses were performed after stratification by tumor type. All tests were two sided and a *p* < 0.05 was considered statistically significant. Analyses were performed using Stata for Windows statistical package (release 13.1, Stata Corporation, College Station, TX).

## Results

### Relationship Between Antibodies and Type of Tumor

**Figure 3:**
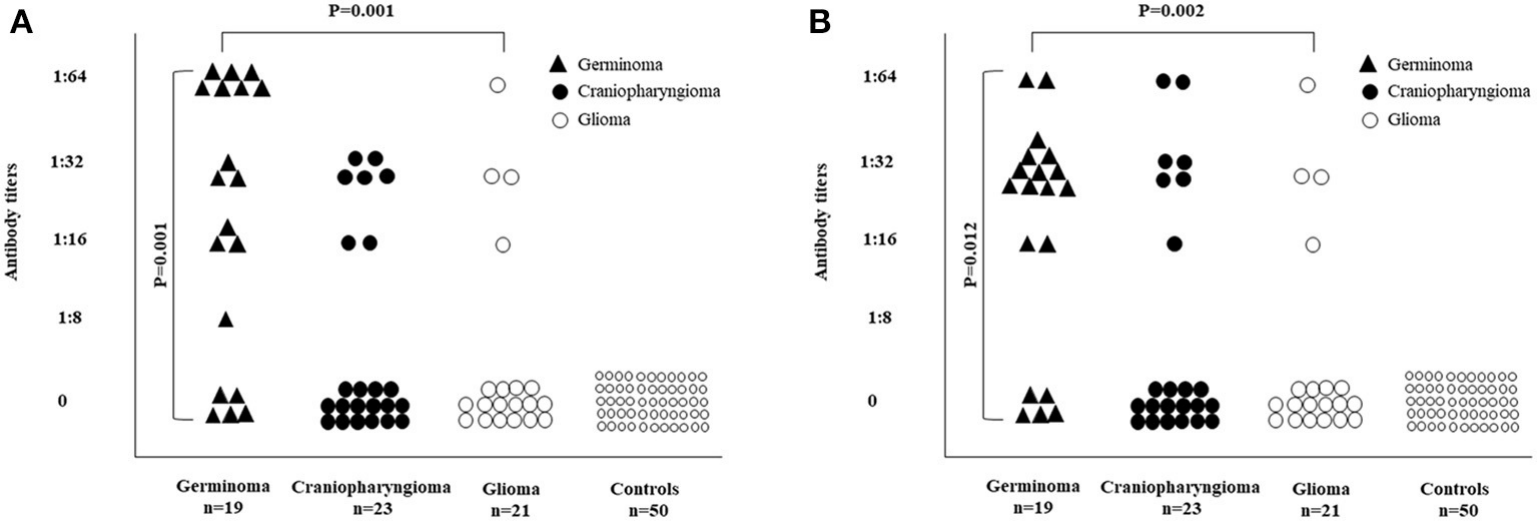
Distribution of APA and AHA in 63 patients with brain tumors according to the diagnostic category. **(A)** The presence of APA (*P* = 0.001) and their titers (*P* = 0.001) were significantly associated with the type of tumor in the following order: germinomas, craniopharingyomas, and gliomas. **(B)** The presence of AHA (*P* = 0.002) and their titers (*P* = 0.012) were significantly associated with the type of tumor in the following order: germinomas, craniopharingyomas, and gliomas.

Among the entire cohort, circulating APA and/or AHA were found in 31 subjects (49.2%) and in none of the healthy controls. In particular, 25 subjects out of 63 were APA positive (39.6%), 26 were AHA positive (41.2%) and 20 were both APA and AHA positive (31.7%). Nine patients with APA and/or AHA had craniopharyngioma (29%), seven (22.6%) had glioma and 15 (48.4%) had germinoma. Patients with craniopharyngioma were positive for at least one antibody in 39.1% compared to 33.3% of the patients with glioma and to 78.9% of those with germinoma with similar distributions for APA and AHA between the three tumors (Table 2). The presence of APA or AHA and of both APA and AHA was significantly increased in patients with germinoma (Table 2). Indeed, the presence of APA (*P* = 0.001) and their titers (*P* = 0.001) were significantly associated with the type of tumor in the following order: germinomas, craniopharyngiomas, and gliomas (Figure 3A); a similar distribution was observed for the presence of AHA (*P* = 0.002) and their titers (*P* = 0.012) (Figure 3B).

**Table 2:** Distribution of anti-pituitary (APA) and anti-hypothalamus (AHA) antibodies based on the type of tumor.

	**Ab** ***n* = 31^**a**^**	**APA** ***n* = 25^**b**^**	**AHA** ***n* = 26^**c**^**	**APA/AHA** ***n* = 20^**d**^**
Craniopharyngiomas (*n* = 23)	9 (29.0)	7 (28.0)	7 (26.9)	5 (21.7)
Gliomas (*n* = 21)	7 (22.6)	4 (16.0)	5 (19.2)	2 (9.5)
Germinomas (*n* = 19)	15 (48.4)	14 (56.0)	14 (53.9)	13 (68.4)

*Ab, At least one antibody positive; APA, only APA positive; AHA, only AHA positive; APA/AHA, both APA and AHA positive*.

a p = 0.007;

b p = 0.001;

c p = 0.002;

d *p = < 0.001*.

### Relationship Between Antibodies and Pituitary Defects

The distribution of APA and AHA based on the type of anterior and posterior pituitary defects is reported in Table 3. The most common anterior and posterior pituitary defect was GH followed by AVP and TSH deficiencies, and a significant association was found between the number of pituitary defects and the presence of APA and/or AHA; in the absence of antibodies, the median number of pituitary defects was 1 (IQR 0–4) compared to 4 in the presence of at least one antibody. In particular, 32 patients among 63 did not have an ACTH deficiency, 9 (28.1%) were AHA positive and 6 (18.7%) were APA positive.

**Table 3:** Distribution of anti-pituitary (APA) and anti-hypothalamus (AHA) antibodies based on pituitary dysfunction.

	**Ab** ***n* = 31**	***p***	**APA** ***n* = 25**	**AHA** ***n* = 26**	**APA/AHA** ***n* = 20**
Number of pituitary defects, Median (IQR)	4 (3–5)	<0.001	5 (4–5)^b^	4 (3.5)^b^	5 (4–5)^b^
AVP (*n* = 39, 61.9%)	25 (64.1)	0.003	22 (56.4)^a^	21 (53.9)^c^	18 (46.2)^a^
GH (*n =* 41, 65.1%)	24 (58.5)	0.043	23 (56.1)^b^	20 (48.8)	19 (46.3)^a^
Pituitary deficits TSH (*n =* 36, 57.1%)	24 (66.7)	0.001	21 (58.3)^b^	21 (58.3)^a^	18 (50.0)^b^
ACTH (*n =* 31, 49.2%)	21 (67.7)	0.004	19 (61.3)^a^	17 (54.8)^c^	15 (48.4)^a^
GN (*n =* 24, 38.1%)	17 (70.8)	0.007	16 (66.7)^a^	14 (58.3)^a^	13 (54.2)^a^

*Ab, At least one antibody positive; APA, only APA positive; AHA, only AHA positive; APA/AHA, both APA and AHA positive; AVP, vasopressin; GH, growth hormone; TSH, thyrotropin-stimulating hormone; ACTH, adrenocorticotropin-stimulating hormone; GN, gonadotropin; IQR, Interquartile range*.

a p < 0.01;

b p < 0.001;

c *p < 0.05*.

### Relationship Between Antibodies, Type of Tumor, and Pituitary Defects

**Figure 4:**
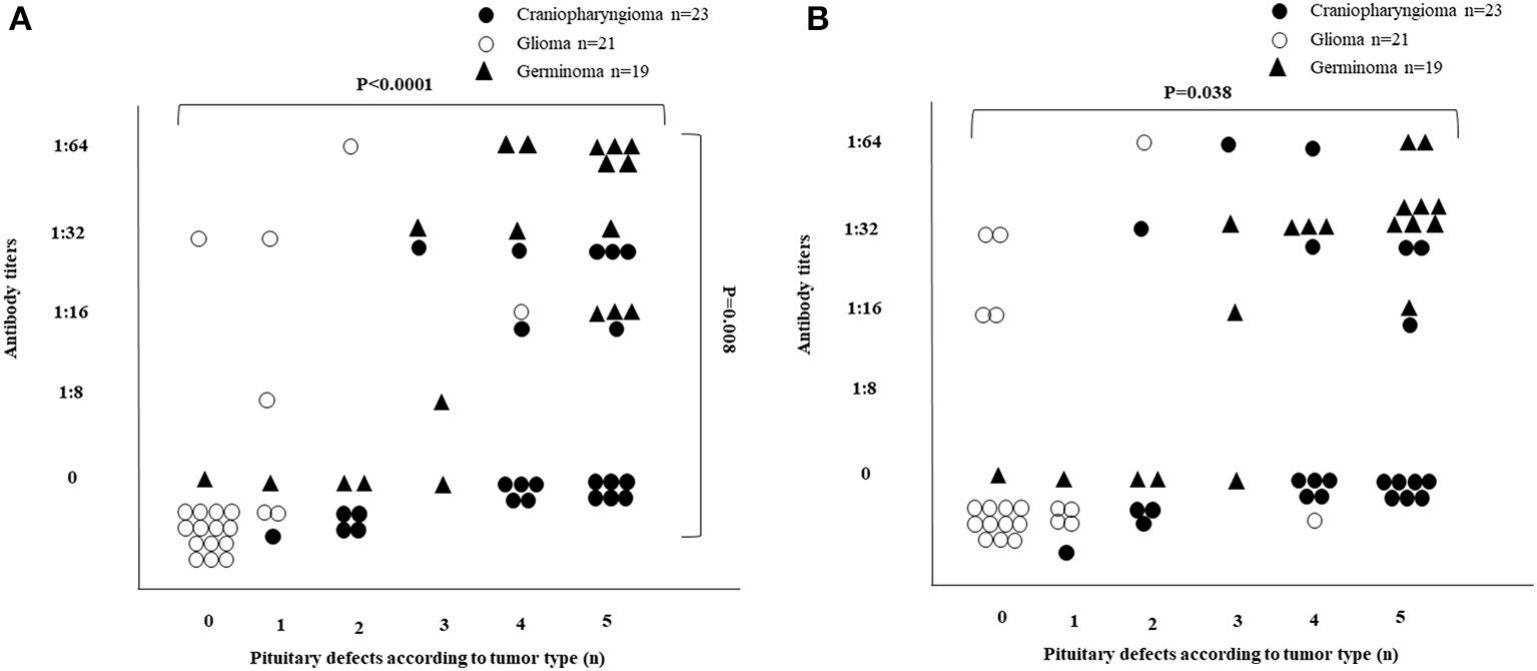
Distribution of APA and AHA in 63 patients with brain tumors based on tumor type and pituitary defects. **(A)** The presence of APA (*P* < 0.0001) and their titers (*P* = 0.008) were significantly associated with the number of pituitary defects (see details in the Results section). **(B)** The presence of AHA was significantly associated with the number of pituitary defects (*P* = 0.038), but not with their titer's level (*P* = 0.145) (see details in the Results section).

The distribution of APA and AHA based on tumor type and pituitary defects are reported in Figure 4. The presence of APA (*P* < 0.001) and their titers (*P* = 0.008) were significantly associated with the number of pituitary defects (Figure 4A). The analysis by type of tumor confirmed such association for gliomas (*P* = 0.023 for the presence of APA, and *P* = 0.013 for their titers, respectively) and for germinomas (*P* = 0.004 and *P* = 0.029, respectively), but not for craniopharyngiomas (*P* = 0.348 and *P* = 0.621, respectively). Poisson regression analysis revealed a statistically significant trend (*P* < 0.001) in the number of defects by APA titers (mean difference percent, MDP, between two APA levels = 24.5%, 95% CI: 13.9–36.2%). This trend was confirmed after adjusting by tumor type (MDP = 18.4%, 95% CI: 6.7–31.5%, *P* = 0.002). The analysis of stratification by tumor type confirmed the same findings in germinomas (MDP = 23.9%, 95% CI: 5.6–45.4%, *P* = 0.009), while the small number of the other patients did not allow their results to be obtained.

Also, the presence of AHA was significantly associated with the number of pituitary defects (*P* = 0.038), but not with their titer's levels (*P* = 0.145) (Figure 4B). A statistically significant association was found among germinomas, limited to the number of pituitary defects (*P* = 0.004), whereas with regard to the titer levels, statistical significance was borderline (*P* = 0.060). No association was found either for craniopharyngiomas (*P* = 0.798 for the number of pituitary defects and *P* = 0.606 for their titers levels) or for gliomas (*P* = 0.271 and *P* = 0.298, respectively). Poisson regression analysis demonstrated a statistically significant trend (*P* < 0.001) in the number of defects by AHA titers (MDP between two AHA contiguous levels = 19.0%, 95% CI: 8.3–30.7%). However, after adjusting by tumor type, such a trend was reduced without statistical significance (MDP = 10.2%, 95% CI: 0.0–22.1%, *P* = 0.061). The analysis of stratification by tumor type revealed a statistically significant trend in the number of defects by AHA titers in germinomas (MDP = 34.1%, 95% CI: 9.4–64.3%, *P* = 0.005).

### Relationship Between Antibodies, Type of Tumor, and Pituitary Defects

**Figure 5:**
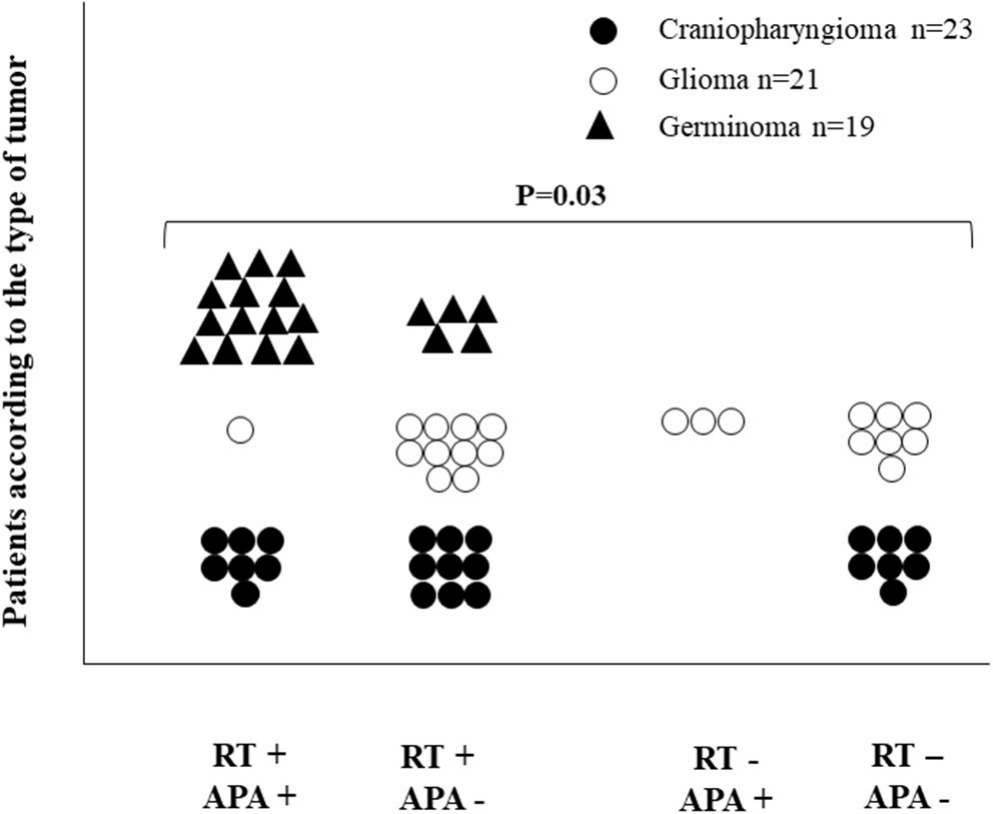
Distribution of 63 patients with brain tumors based on tumor type, presence of APA, and radiotherapy. APA and radiotherapy were significantly associated (*P* = 0.03).

The distribution of patients with brain tumors based on tumor type, presence of APA, and radiotherapy are reported in Figure 5. APA and radiotherapy were significantly associated (*P* = 0.03). According to the type of tumor, such an association was observed among the craniopharyngioma group, with a borderline statistical significance (*P* = 0.057), while no association was observed with gliomas (*P* = 0.311).

### Relationship Between Antibodies and Other Factors

No significant decrease in the presence of APA and AHA and their titers was observed since the time of tumor diagnosis (*P* = 0.88 for APA and *P* = 0.67 for AHA). There was no significant gender differences neither in the presence or absence of antibodies (*P* = 0.79 for APA and *P* = 0.98 for AHA) nor in the titer levels (*P* = 0.85 for APA and *P* = 0.58 for AHA); no significant associations were found between the presence of APA and/or AHA (*P* = 0.96 for APA and *P* = 0.22 for AHA) or their titers (*P* = 0.58 for APA and *P* = 0.57 for AHA) with chemotherapy.

## Discussion

The identification of APA and AHA in different conditions affecting the HP region and their role was poorly investigated in the pediatric age so far, while the limited number of patients studied with the non-specificity of inclusion criteria led to conflicting results (23–33). As in our previous study, we found that one among two patients with germinoma had AHA shortly after the diagnosis (13), we aimed to evaluate systematically the expression of antibodies against the hypothalamus and pituitary in children and adolescents with childhood-onset intracranial tumors. In particular, we attempted to detect the presence of APA and AHA after the diagnosis of craniopharyngioma, glioma, or germinoma, and to explore their relationship with pituitary function.

Our findings indicate that antibodies against pituitary or hypothalamic cells are detectable in patients with childhood-onset brain tumors. In particular, we identified a high incidence of APA and AHA autoantibodies in approximately 50% of our cohort, which was significantly higher than previously reported in different conditions (25–33). In addition, around 79% of our patients with germinoma were positive for at least one of the antibodies compared to 40 of the patients with craniopharyngioma and one third of those with glioma. The presence of antibodies in our patients—and their absence in the controls—supports the hypothesis that HP involvement by a host reaction to the tumor could be responsible for the local autoimmunity and of a consequent potential damage. The analysis of antibody distribution among patients stratified by the tumor type showed that those with germinoma carried the highest prevalence of APA and AHA with no significant difference between the two antibodies. In particular, patients with germinoma had a prevalence of APA/AHA antibodies two to three times higher than those with craniopharyngioma and up to more than six times higher than those with a glioma, respectively, suggesting that germinomas are associated with more susceptibility of HP tissues to autoimmune reaction, the role of which needs further investigation. Our study differs from those that evaluated the presence of HP antibodies in adults with undefined sellar or parasellar lesions or with secreting and non-secreting adenomas, in which the main discrepancies are related to the different time/age of APA antibody detection (23, 24). Hence, the incidence of APA and AHA in our patients with germinoma deserves special attention since systemic and pituitary autoimmunity (34) and lymphocyte infiltration that mimic autoimmune infundibulo-neurohypophysitis or pituitary hypophysitis were reported in patients who were later diagnosed with germinoma (1–3, 34–39). Moreover, in most cases with germinoma (75%), the infiltrating lymphocytes are seen side by side to the nests of neoplastic cells, establishing a two-cell model; in the remaining minority (25%), the lymphocytes largely exceed some isolated neoplastic cells, which therefore become much more difficult to identify (40).

A high number of pituitary deficiencies were found in patients with APA and/or AHA compared to those without antibodies; only 22% of the patients with glioma have APA and/or AHA with most of them without endocrine defects or mainly with a single pituitary defect. In fact, more than 60% of the patients with antibodies belong to the craniopharyngioma and germinoma group and have at least three hormone defects with further increase in the number of pituitary deficiencies in those with both APA and AHA. An interesting point emerging from our data is the high prevalence of isolated or multiple pituitary/hypothalamic hormone deficiency in patients with APA and/or AHA positive at a high titer. This seems to indicate that these antibodies may be directed to one or more pituitary- or hypothalamic-secreting cells. The clinical significance of the APA titer in patients is a further point to be discussed. Although in our previous studies on APA detection where the interpretation could be subjective, an arbitrary cutoff for positivity of 1:8 was fixed to discriminate the true from the possible false-positive results. We considered for our analysis only the patients positive for APA and AHA at a high titer (>1:32); in addition, the antibodies were evaluated by two different operators in a double-blind manner. Thus, we proposed that APA should be considered effective diagnostic markers of autoimmune pituitary dysfunction only when detected at high titer (23). However, an appropriate study using a double immunofluorescence method is needed and is in progress to clarify which cells are the target of these antibodies. The estimates of association between antibody titers and the number of pituitary defects, indeed, should be considered with some caution, due to the semiquantitative nature of the measurement method employed (immunofluorescence). However, even if the assumption of a linear relationship might not be completely met, the Poisson regression analysis revealed a statistically significant trend in the number of defects by APA titer levels, suggesting that each increase in antibody titers from one level to a higher one was associated with a 25% increased risk of developing an additional endocrine defect, and that this increase was particularly significant in germinomas either for APA (23.9%) and AHA (34.1%) titer's level.

The association of MPHD with APA and or AHA both in craniopharyngiomas and germinomas suggest that these findings cannot be considered incidental, but the result of a possible association, thus raising the question: Why can two tumors cause a similar endocrine dysfunction, but different frequency of autoantibodies? The presence of APA and/or AHA with a different incidence in the three tumors indicates that their position at the hypothalamic level can lead to local autoimmune reactions, though we believe that other factors must be involved, including intrinsic destructiveness of these tumors and their treatment regimens. In fact, no significant associations were found between the presence of APA and/or AHA or their titers with chemotherapy, but a significant association of APA with radiotherapy was observed. Our findings suggest that APA and/or AHA may represent markers of extension/severity of the hypothalamic–pituitary host reaction to the tumor (represented by the infiltration of prominent lymphocytes) and a response to autoantigen exposure with consequent stimulation of an autoimmune process. Germinoma involving the pituitary gland and the hypothalamus could trigger the main immune response, while the absence of the blood–brain barrier at the median eminence represents the diffusion pathway of the antibodies, with consequent further impairment of the pituitary function. As far as radiotherapy is concerned, its potential confounding role, or additional contributory effect to the pituitary damage, cannot be ruled out because the antibodies were tested at a single time point in a cross-sectional study.

In summary, our results suggest that brain tumors in children and adolescents are associated with the development of hypothalamic–pituitary antibodies and pituitary defects. In addition, the association between APA and brain tumor-induced hypopituitarism may provide a new point of view in this field and promote further clinical and experimental studies. Patients with germinoma are prone to develop an autoimmune process that contributes to endocrine dysfunction, while the type and pattern of antibodies could be influenced by therapeutic regimens. The long-term evaluation of additional damage in patients with partial pituitary dysfunction remains to be determined, while attention should be paid to the interpretation of APA/AHA antibodies in order to avoid missing the diagnosis of germinomas masked by an autoimmune pituitary condition.

## Data Availability Statement

All datasets generated for this study are included in the article/supplementary material.

## Ethics Statement

All procedures performed in this study were in accordance with the ethical standards of the institutional and/or national research committee and with the 1964 Helsinki declaration and its later amendments or comparable ethical standards.

## Consent for Publication

Informed consent was obtained from all individual participants who were included in the study.

## Author Contributions

GP and EC followed-up the patients, collected the data, and drafted and revised the manuscript. AD, GB, and MIM performed antibody measurements and revised the manuscript. FN and AA took care of the patients and revised the manuscript. AG, HT, and MC helped in the evaluation and the following-up of the patients. MG was the neuro-oncologist who took care of the patients. SP performed the statistical analyses. MM designed the study and actively participated in drafting and revising of the manuscript. NI designed the study, supervised the patients, and actively participated in data analysis, drafting, and revision of the manuscript. All authors approved the final manuscript as submitted and agree to be accountable for all aspects of the work.

### Conflict of Interest

The authors declare that the research was conducted in the absence of any commercial or financial relationships that could be construed as a potential conflict of interest.
